# Novel Gd^3+^-doped silica-based optical fiber material for dosimetry in proton therapy

**DOI:** 10.1038/s41598-019-52608-5

**Published:** 2019-11-08

**Authors:** C. Hoehr, A. Morana, O. Duhamel, B. Capoen, M. Trinczek, P. Paillet, C. Duzenli, M. Bouazaoui, G. Bouwmans, A. Cassez, Y. Ouerdane, A. Boukenter, H. El Hamzaoui, S. Girard

**Affiliations:** 1 TRIUMF, 4004 Wesbrook Mall, Vancouver, Canada; 2 Univ-Lyon, Laboratoire H. Curien, F-42000 Saint-Etienne, France; 3 CEA, DAM, DIF, Arpajon, F-91297 France; 4 Univ-Lille, UMR 78523-PhLAM – Physique des Lasers, Atomes et Molecules, F-59000 Lille, France; 5 British Columbia Cancer, Vancouver, Canada; 6 University of British Columbia, Physics and Astronomy, Vancouver, Canada

**Keywords:** Materials for optics, Optical physics

## Abstract

Optical fibers hold promise for accurate dosimetry in small field proton therapy due to their superior spatial resolution and the lack of significant Cerenkov contamination in proton beams. One known drawback for most scintillation detectors is signal quenching in areas of high linear energy transfer, as is the case in the Bragg peak region of a proton beam. In this study, we investigated the potential of innovative optical fiber bulk materials using the sol-gel technique for dosimetry in proton therapy. This type of glass is made of amorphous silica (SiO${}_{2}$) and is doped with Gd${}^{3+}$ ions and possesses very interesting light emission properties with a luminescence band around 314 nm when exposed to protons. The fibers were manufactured at the University of Lille and tested at the TRIUMF Proton Therapy facility with 8.2–62.9 MeV protons and 2–6 nA of extracted beam current. Dose-rate dependence and quenching were measured and compared to other silica-based fibers also made by sol-gel techniques and doped with Ce${}^{3+}$ and Cu${}^{+}$. The three fibers present strong luminescence in the UV (Gd) or visible (Cu,Ce) under irradiation, with the emission intensities related directly to the proton flux. In addition, the 0.5 mm diameter Gd${}^{3+}$-doped fiber shows superior resolution of the Bragg peak, indicating significantly reduced quenching in comparison to the Ce${}^{3+}$ and Cu${}^{+}$ fibers with a Birks’ constant, k${}_{B}$, of (0.0162 $\pm$ 0.0003) cm/MeV in comparison to (0.0333 $\pm$ 0.0006) cm/MeV and (0.0352 $\pm$ 0.0003) cm/MeV, respectively. To our knowledge, this is the first report of such an interesting k${}_{B}$ for a silica-based optical fiber material, showing clearly that this fiber presents lower quenching than common plastic scintillators. This result demonstrates the high potential of this inorganic fiber material for proton therapy dosimetry.

## Introduction

Optical fiber detectors have been attracting increased attention in radiotherapy dosimetry^1–10^, particularly for small fields due to their enhanced spatial resolution. Next to traditional radiotherapy with high energy photons, radiotherapy with protons is becoming increasingly available with currently 86 hadron therapy centers in operation and 71 being planned and built^11^. Protons can be advantageous for some cancer sites due to their depth-dose profile exhibiting a sharp peak and a steep dose gradient at the end of their range (Bragg peak). The Markus chamber, a small parallel-plate ionization chamber, is the gold standard for measurement of the depth-dose deposition in proton therapy. However, its extended size in the dimension perpendicular to the beam direction hampers its ability to measure dose in the mm range. Optical-fiber detectors can be sub-mm in diameter and made of either organic or inorganic materials. Organic-based materials have the advantage of being essentially water-equivalent whereas inorganic-based materials (often amorphous silica glasses) have the advantage of greater light yield and radiation hardness when appropriate dopants are selected.

A detector for radiotherapy dosimetry should have a response that depends linearly on dose and is independent of both energy and dose rate, as well as possessing superior spatial resolution. Since the linear energy transfer (LET) does change dramatically along the Bragg peak of proton therapy, the independence of both energy and dose rate are crucial characteristics in any new detector. Most scintillation detectors are known to exhibit increased quenching when LET increases^4,6,12–17^ due to the participation of alternate modes of energy dissipation in regions of high ionization density. In order to resolve the Bragg peak of the proton dose depth profile, a fiber diameter of 250 $\mu$m or less may be required^12^. If the fiber is larger in diameter, a volume-averaging effect will result in a broader and lower Bragg peak.

In this paper the response of a novel silica-based sol-gel glass, coupled to an optical transport fiber and doped with Gd${}^{3+}$ rare-earth ions, to radiation-induced luminescence (RIL) is investigated for applications in proton therapy. Energy dependence and dose-rate dependence for $\sim$ 500 $\mu$m diameter glassy rods attached to 500 $\mu$m fibers are measured and compared to the Markus chamber as well as to Ce${}^{3+}$- and Cu${}^{+}$-doped fibers. The proton irradiation results of our Cu${}^{+}$- and Ce${}^{3+}$-doped fibers are described elsewhere^3,14^.

## Materials and Methods

### Fibers

Gd${}^{3+}$-, Ce${}^{3+}$-, or Cu${}^{+}$-doped silica fibers were all prepared using the same sol-gel technique and the same drawing facilities at PhLAM Laboratory and FiberTech Lille platform of the University of Lille. Porous silica xerogels were prepared, using the sol-gel technique from tetraethylorthosilicate (TEOS) precursor as described elsewhere^18^. Before the incorporation of dopants, using a solution doping technique, the obtained xerogels were stabilized at 1000 ${}^{\circ }$C^19^. These xerogels were soaked in an alcoholic solution containing dopant salts. Subsequently, the samples were withdrawn from the doping solution and dried at 50 ${}^{\circ }$C for 24 hours to remove solvents and to retain the dopant element within the nanopores. The doped matrices were then densified in a helium atmosphere for 2 hours^20–23^. Finally, the Gd${}^{3+}$-, Ce${}^{3+}$-, or Cu${}^{+}$-doped glassy rods were drawn at a temperature of about 2000 ${}^{\circ }$C down to a millimeter-sized cane. The concentrations of dopant in the cane samples were estimated using electron probe microanalysis (EPMA) at about 0.1 wt% and 0.07 wt% for Gd and both Ce and Cu, respectively.

### Radioluminescence

To measure the radioluminescence of the Gd${}^{3+}$-doped fiber, irradiation tests have been performed with the MOPERIX machine of the University of Saint-Etienne which is equipped with a tungsten target. The sample under test has been exposed to an x-ray beam, having a mean energy of 40 keV at a dose rate of 10 Gy(SiO${}_{2}$)/s. The resulting spectrum was recorded by connecting the fiber to a spectrometer (QE Pro Ocean optics).

### Proton Irradiations

**Figure 1:**
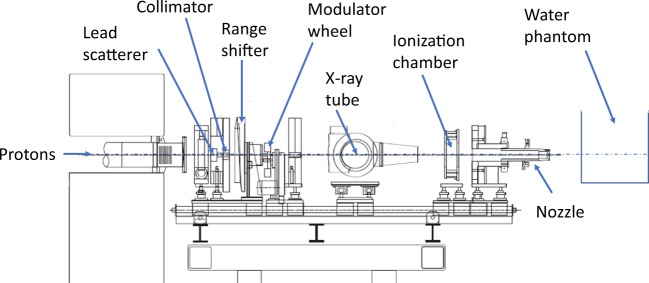
Schematic of the beamline. The water phantom and detectors are positioned distal to the nozzle. The front surface of the water phantom is located 70 mm downstream of the nozzle output. Note that the x-ray tube is used for image-guided patient setup and translates out of the beam line during beam delivery.

Proton irradiations were performed at the Proton Therapy facility at TRIUMF^24^, the only Proton Therapy centre in Canada to date^25,26^, and one of several centers specializing in treating ocular cancer worldwide^27^. The facility consists of a dedicated horizontal beam line, extracting 74 MeV protons from the TRIUMF main cyclotron^28^. The beam delivery system is passive, see Fig. 1: The incoming proton beam first intercepts a 0.8 mm-thick scatterer of lead to spread the beam in size and then passes through a collimator of brass to limit the proton beam to an almost flat profile. The proton beam is then degraded to the required maximum energy using a range shifter made from PMMA plastic. The proton beam is then spread axially to fully cover the tumor volume using a stepped, rotating PMMA modulator wheel. Lastly, a patient-specific collimator of brass at the nozzle output is used to conform the proton beam to the transverse tumor shape. For this study, we used modulator wheels to produce 23 mm spread-out Bragg peaks (SOBP), and a circular brass collimator of 25 mm diameter was employed. The delivered dose to a phantom or patient is monitored by an ionization chamber just upstream of the treatment nozzle, calibrated using the IAEA protocol^29^.

**Figure 2:**
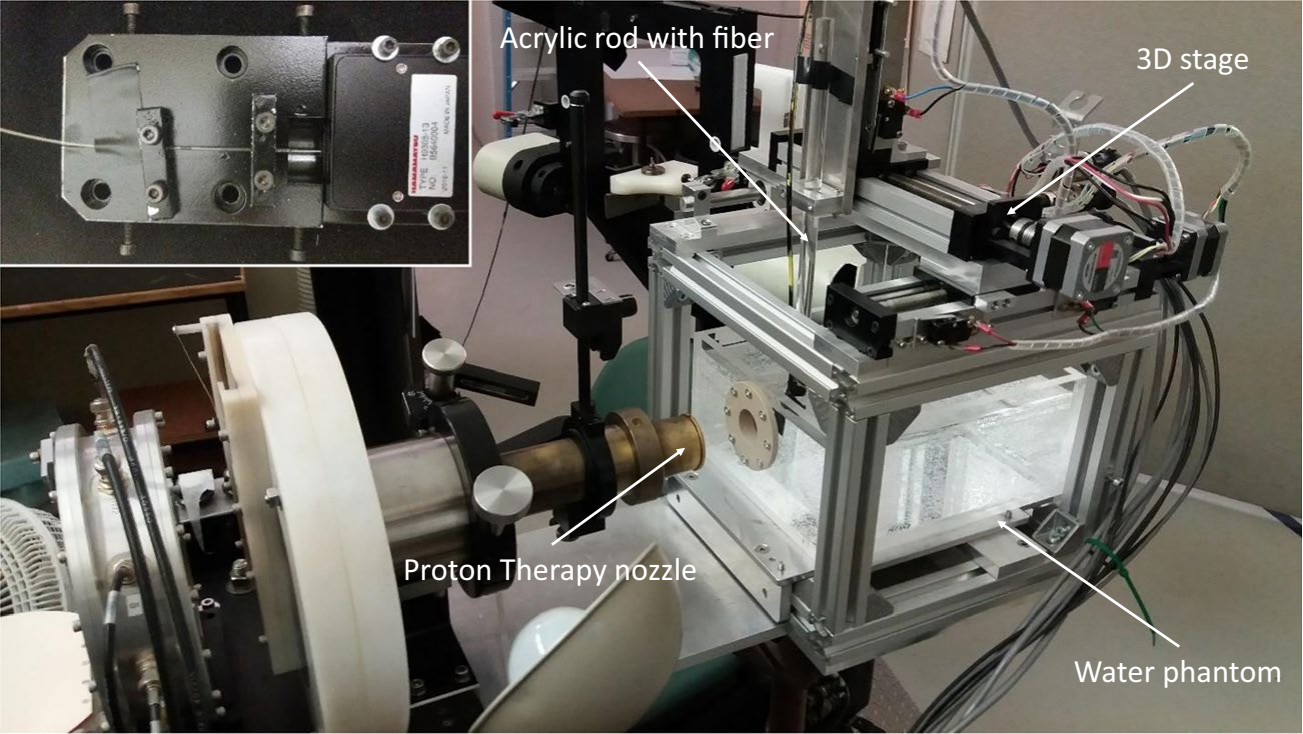
Experimental setup. The proton beam is coming from the left through the proton therapy nozzle before entering the water phantom through the solid-water window. The fiber is attached to the acrylic rod, mounted on the 3D stage. Linear stages in three directions allow the fiber to be moved remotely through the water phantom. Inset: The fiber is guided to the PMT via a custom-made box, shown here with the light-tight cover removed.

For RIL measurements, the experimental setup was composed of a 0.5 mm-diameter $\times$ 10 mm-length piece of the Gd${}^{3+}$-, Cu${}^{+}$- or Ce${}^{3+}$-doped cylindrical cane, one end of which was fusion-spliced to a 5 m-long multimode silica fiber. This transport optical fiber, with a core diameter of 0.5 mm coated with a low refractive index polymer cladding, was used to guide the RIL signal toward a photomultiplier module for detection. To reduce background signal and noise from ambient light, the fibers were sheathed in black tubing and the lighting in the treatment room was switched off. The doped end of the fiber optic was connected to an acrylic rod mounted on a 3D stage using black electrical tape to further enhance light-tightness and then inserted into the water phantom commonly used for clinical calibrations, see Fig. 2.

The water phantom has a 1 mm-thick water equivalent entrance portal to ensure that different detectors can be positioned to within 1 mm water equivalent depth of the surface. The mounting rod can be remotely moved in all three dimensions in sub-mm increments via stepper motors. The output end for the fiber optic was sent to a light-tight box housing a compact and low-cost photomultiplier tube (PMT, Hamamatsu, model H9305-13). The analog signal from the PMT was then either directly recorded with a numerical oscilloscope (Tektronix) or first amplified by an electrometer (Wellhofer, WP5007) and then further processed using the TRIUMF in-house data acquisition system. This system integrates the measured current for a prescribed dose as determined by the monitor counts (MC) of the in-air ionization chamber of the proton beam line.

Quenching was estimated by measuring the depth dose along the beam axis in the water phantom and comparing with the Markus chamber depth dose curve. The depth dose was measured as a raw Bragg peak with a single energy proton beam of 74 MeV at extraction, and a spread-out-Bragg peak (SOBP). To spread the Bragg peak, a PMMA modulator wheel is placed in the beam path and rotated with a frequency of 240 rpm. The wheel consists of steps of different thicknesses. Each of these steps modulates the beam energy, creating Bragg peaks at different depths in the water phantom (or patient). When all the Bragg peaks are added together, the result is a flat SOBP. The Markus chamber measures dose in air, while the three fibers measure dose in silica.

Dose-rate dependence was measured at different energies ranging from 8.2 MeV to 62.9 MeV by placing the fiber at the front of the water phantom and degrading the proton beam upstream of the phantom by inserting PMMA of different thicknesses via the range shifter while a raw Bragg peak of 70 MeV was delivered. The proton beam current was varied from 2 nA to 6 nA. From this, a dose rate and a proton flux rate were determined from facility calibrations.

### Quenching correction factor

Birks^30^ modelled to first order the quenching of a scintillator as a function of LET. Chou^31^ and Craun and Smith^32^ added a second order relationship, as given by Equation 1,

1 $$\frac{dL}{dx}=\frac{S\frac{dE}{dx}}{1+{k}_{B}\frac{dE}{dx}+C{(\frac{dE}{dx})}^{2}}$$

where $L$ is the emitted fluorescence signal of the scintillator, $S$ is the scintillation efficiency, $dE/dx$ is the proton stopping power (and $E$ is the energy deposited by the particle), ${k}_{B}$ is Birks’ constant and $C$ is an additional parameter which may be used to obtain an improved fit. The Monte-Carlo package SRIM^33^ was used to calculate the LET as a function of depth for the raw 74 MeV Bragg peak. Quenching was assessed by normalizing the doped fiber’s RIL signal for LET = 15 MeV/cm${}^{2}$ to be consistent with the work of Torrisi *et al*.^10^ and the resulting signal was plotted against the LET and fitted using Equation 1 to determine k${}_{B}$.

## Results and Discussion

### Radioluminescence

**Figure 3:**
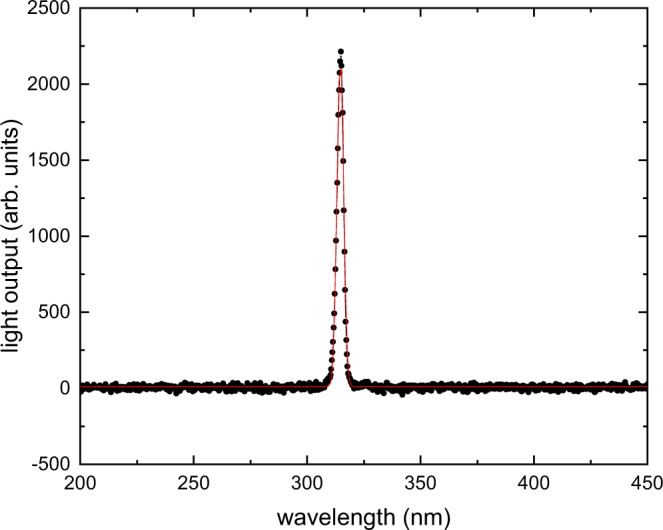
Radioluminescence spectrum of the Gd${}^{3+}$-doped fiber in response to x rays with a mean energy of 40 keV. The peak centers at (314.61 $\pm$ 0.01) nm with a FWHM of (3.41 $\pm$ 0.03) nm, as determined by a Gaussian fit (solid line).

Figure 3 shows the radioluminescence spectrum of the Gd${}^{3+}$-doped material. Due to the electronic structure (4f${}^{7}$) and consequent energy levels in the Gd${}^{3+}$-doped amorphous silica, only one line at (314.61 $\pm$ 0.01) nm with a FWHM of (3.41 $\pm$ 0.03) nm is observed. When the Gd${}^{3+}$ ion occupies a non centrosymmetric site in solids, it is known that transitions between Gd${}^{3+}$-energy levels have a mixed forced electric dipole-magnetic dipole character^34,35^. The observed band corresponds to the ${}^{6}$P${}_{7/2}$ $\to$ ${}^{8}$S${}_{7/2}$ transition of the Gd${}^{3+}$ ion. This transition has a mixed forced electric dipole-magnetic dipole character since in silica glasses, the Gd${}^{3+}$ ion, as other rare-earth ions, occupies a low-symmetry site^36^. It can also be noticed that the FWHM of the band assigned to the ${}^{6}$P${}_{7/2}$ $\to$ ${}^{8}$S${}_{7/2}$ transition is smaller in comparison to those encountered for other rare-earth ions in solids. It was already reported in the literature that neither emission nor absorption bands of Gd${}^{3+}$ in glasses show any resolved structure due to the Stark splitting induced by the crystal field. Furthermore, the corresponding bands are significantly narrower than those associated with other rare-earth ions in the same material^34^. This observation is explained by the fact that, for Gd${}^{3+}$, the crystal field effect is even lower than for other trivalent rare-earth ions as a consequence of its electronic structure (4f${}^{7}$)^37,38^.

### Dose rate dependence

**Figure 4:**
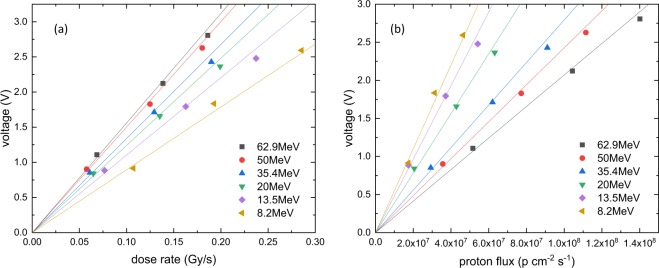
(**a**) Dose-rate dependence of the output voltage of the Gd${}^{3+}$-doped fiber for different proton energies. (**b**) Proton flux dependence of the output voltage of the Gd${}^{3+}$-doped fiber for different proton energies. For both figures, the lines are linear fits going through the origin.

**Figure 5:**
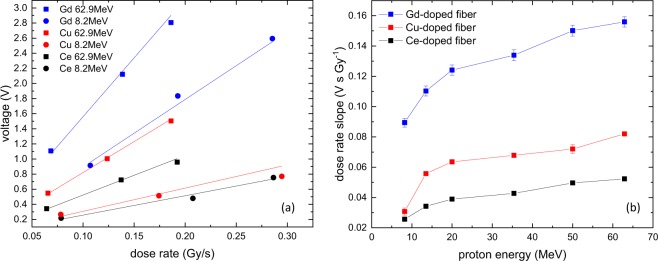
(**a**) Dose-rate dependence for the smallest and largest incident proton energy for all three fibers. (**b**) Dose rate slope evolution versus the incident proton energy for the three studied fibers.

The effect of changing beam intensity, expressed as dose-rate dependence and proton flux dependence is shown in Fig. 4. For a given proton energy, the dependence is linear, with different slopes for different energies. This change in slope for the dose-rate dependence is shown in Fig. 5, compared with the Cu${}^{+}$- and Ce${}^{3+}$-doped fiber results. From this graph, it is clear that the Gd${}^{3+}$-fiber has the largest and the Ce${}^{3+}$-doped fiber the smallest signal and slopes, or dose-rate dependence. For all three fibers, the change in slope is smaller for higher energies above 20 MeV and larger for smaller energies due to the higher LET in this range, which accelerates the energy dependence.

### Quenching

**Figure 6:**
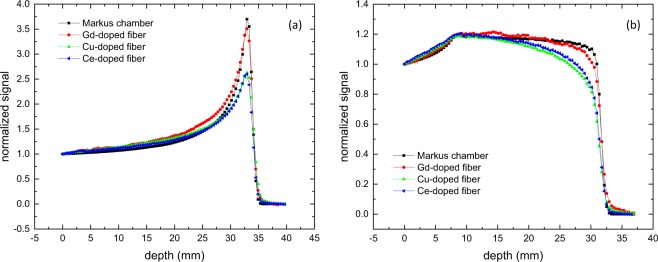
(**a**) Depth-dose profile of a raw-Bragg peak of a 74 MeV proton beam deposited in a water phantom, normalized to the entrance signal. The peak-to-entrance ratio of the Markus chamber in air is 3.7. For all three fibers (silica), the peak-to-entrance ratio of the Gd${}^{3+}$-doped fiber is 3.5, Cu${}^{+}$-doped fiber 2.6 and Ce${}^{3+}$-doped fiber 2.6. (**b**) Depth-dose profile of a 23 mm SOBP with a 74 MeV proton beam in a water phantom, normalized to the entrance signal.

The measured depth-dose profile for 74 MeV protons and a 23 mm SOBP up to 74 MeV with the Markus chamber and the three fibers are shown in Fig. 6. All four data sets are normalized at the water-phantom entrance. To investigate the spatial resolution of the detectors, the distal fall-offs were fitted with a Boltzmann equation. The resulting fit function was then used to determine the 80% and the 20% height of the distal fall off. As expected, the Markus chamber measures the highest peak-to-entrance ratio of 3.7, due to its minimal quenching at high LET and very small sensitive volume in the axial direction. This small volume results in a depth resolution of the distal fall-off of 0.75 mm, measured as the distance between the 80% height and the 20% height. This spread in depth represents the energy spread of the proton beam of 1 MeV at extraction and the resolution of the measurement technique. The Gd${}^{3+}$-doped fiber still has an excellent peak-to-entrance ratio of 3.5, while both the Cu${}^{+}$- and Ce${}^{3+}$-doped fibers exhibit severe quenching with a peak-to-entrance ratio of only 2.6 for both. Due to the 0.5 mm diameter of all three fibers the 80%–20% depth resolution is 0.93 mm for the Gd${}^{3+}$ fiber, 1.05 mm for the Cu${}^{+}$ fiber and 0.95 mm for the Ce${}^{3+}$-doped fiber. The effect of the quenching is shown in Fig. 6(b). With minimal quenching, the SOBP is measured as a flat plateau with a clearly defined distal fall-off. The Gd${}^{3+}$-doped fiber has a slightly more sloped plateau and more rounded fall-off. Both the Cu${}^{+}$- and Ce${}^{3+}$-doped fibers show a clear slope and it is very difficult to determine the fall-off of the curve and with it the range of the SOBP. With such characteristics, these two fibers would not be suitable for clinical proton dosimetry.

It should be noted that a fiber with a diameter of 0.5 mm will not fully resolve the raw Bragg peak as discussed by Archambault *et al*.^12^, and this effect may be confounded with true signal quenching. All three doped fibers were 0.5 mm in diameter, allowing comparisons on the basis of quenching alone between each other. To estimate the effect of the extended size, the data from the Markus chamber was convoluted with a Gaussian distribution with a standard deviation of 0.25 mm. This results in broadening of the Bragg peak measured with the Markus chamber to a 80-20 % distance of 0.86 mm, and lowering the peak-to-entrance ratio to 3.57, much closer to the Gd${}^{3+}$-doped fiber with a ratio of 3.51. This convolution is just a rough estimation as it does not take into account that the Markus chamber measures dose in air, while all three fibers measure dose in silica.

**Figure 7:**
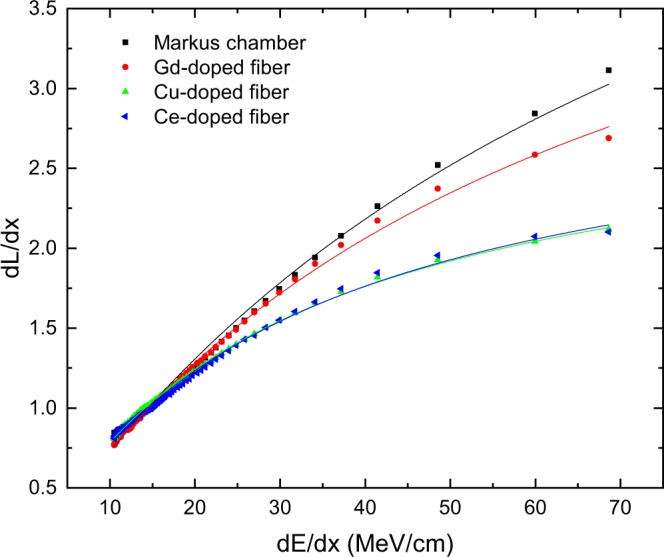
Light emission $dL/dx$ as a function of $dE/dx$ for a pristine Bragg peak at 74 MeV proton energy. The data sets are normalized at $dE/dx$ = 15 MeV/cm to be consistent with Torrisi *et al*.^10^. Solid lines are a fit with the Birks equation 1. k${}_{B}$ for the Markus chamber is k${}_{B}$= (0.0124 $\pm$ 0.0005) cm/MeV, for the Gd${}^{3+}$-doped fiber k${}_{B}$= (0.0162 $\pm$ 0.0003) cm/MeV, for the Cu${}^{+}$-doped fiber k${}_{B}$= (0.0352 $\pm$ 0.0003) cm/MeV, and for the Ce${}^{3+}$-doped fiber is k${}_{B}$= (0.0333 $\pm$ 0.0006) cm/MeV. Best fit results were achieved with $C$ = 0.

To further quantify the detector response, we plotted the fiber signals against LET and fitted equation 1. The result is shown in Fig. 7. While the Markus chamber curve has a k${}_{B}$= (0.0124 $\pm$ 0.0005) cm/MeV and the Gd${}^{3+}$-doped fiber has still a very good k${}_{B}$= (0.0162 $\pm$ 0.0003) cm/MeV. On the other hand, the Cu${}^{+}$- and Ce${}^{3+}$-doped fibers have k${}_{B}$= (0.0352 $\pm$ 0.0003) cm/MeV and k${}_{B}$= (0.0333 $\pm$ 0.0006) cm/MeV, respectively. These values for the Cu${}^{+}$- and Ce${}^{3+}$-doped fibers are in line with results from Savard *et al*.^15^ and Hoehr *et al*.^4^ for a Ce${}^{3+}$-doped fiber and a plastic scintillator fiber presenting a Birks’ constant around 0.0305 $\pm$ 0.0006 cm/MeV. Besides the fact that inorganic materials such as the Gd${}^{3+}$-doped glass present generally increased radiation hardness, the Gd${}^{3+}$-doped fiber also shows a lower Birks constant, meaning that this fiber experiences lower quenching than common plastic scintillators and Cu${}^{+}$- and Ce${}^{3+}$-doped silica-based fibers. It should be noted that other dosimeters, like for example the PRESAGE^39^ can exhibit even lower levels of quenching.

As seen earlier, the material has a single emission line at around 314 nm under excitation with a 40 keV average energy x-ray beam and no significant alternate optical mode of energy dissipation. Its unique sharp emission line suggests that quenching through other optical energy dissipation modes is unlikely compared with other scintillating materials which generally have a broader emission spectrum^20,21^. Indeed, it is known that the energy transfer depends on many physical parameters, such as distance between the donor and the acceptor, the concentrations of donors and acceptors, and the spectral overlap between the donor emission spectrum and the acceptor absorption. Higher degrees of the spectral overlap between the donor emission spectrum and the acceptor absorption spectrum yield greater energy transfer efficiency. Generally, defects in silica glasses presents absorption bands with typical bandwidths of several tens of nanometers (such as ODC or NBOHC defects) and thus, the spectral overlap between these absorption bands and the sharp emission line of Gd${}^{3+}$ is lower than with broader emissions of Cu${}^{+}$ and Ce${}^{3+}$ ions, leading to a low energy transfer. This aspect may constitute one of the reasons that contributes to the reduction of the quenching phenomena in the case Gd${}^{3+}$-doped fiber. Another aspect that could also play an important role in the decrease of the quenching is the absence of residual quenching centers with Gd${}^{3+}$ ions. In the case of Ce${}^{3+}$- and Cu${}^{+}$-doped fibers, despite the helium atmosphere heat-treatment, which is applied to increase the PL efficiency^20–22^, silica glasses still contain Ce${}^{4+}$ and Cu${}^{2+}$ ions, respectively. The presence of these optically inactive ions could explain the higher quenching effect observed under proton exposure in comparison to Gd${}^{3+}$-doped sample. Concerning Cu-doped glass, this effect can be explained by energy transfer from Cu${}^{+}$ ions to residual Cu${}^{2+}$ ions, which then relax non radiatively^40^. Moreover, Ce${}^{4+}$ ion, owing to its strong charge transfer (CT) absorption can act as a quenching center for the Ce${}^{3+}$ luminescence^41^. Besides, as the protons could generate neutrons that participate to the RIL process inside the doped glass, the neutron absorption cross sections of dopant elements should have an impact. According to the literature^42^, Gd presents the highest neutron absorption cross sections compared to both Ce and Cu. The corresponding absorption cross sections are of about 242000, 0.66 and 4.5 barn for Gd, Ce and Cu, respectively. This might also explain the low quenching effect observed with Gd. Gd${}^{3+}$-doped silica glass hence demonstrates significantly less quenching in proton beams in comparison to other scintillation detectors described to date.

## Summary and Outlook

We present a novel Gd${}^{3+}$-doped fiber material for use in proton therapy dosimetry. The fiber is a very promising candidate due to its small size (500 $\mu$m diameter), its favorable dose-rate dependency and its weak dependency on proton energy. This last point is especially important as this reduces the quenching effect in regions of high LET, i.e. the Bragg peak. The size of the 0.5 mm diameter detector may result in the Bragg peak not being fully resolved spatially and volume averaging may be confounding the characterization of quenching alone. However, the Birks constant k${}_{B}$ is estimated to be at the most k${}_{B}$= (0.0162 $\pm$ 0.0003) cm/MeV, not much higher than for the clinically used Markus chamber with k${}_{B}$= (0.0124 $\pm$ 0.0005) cm/MeV. In comparison, the Birks constant is k${}_{B}$ =  (0.0352 $\pm$ 0.0003) cm/MeV and k${}_{B}$= (0.0333 $\pm$ 0.0006) cm/MeV for Cu${}^{+}$- and Ce${}^{3+}$-doped fibers, respectively. In addition, the Gd${}^{3+}$-doped material exhibits a high sensitivity level as well as a stable response after several proton irradiations.

As recommended by Archambault *et al*.^12^, a detector diameter of 250 $\mu$m or less may address the volume averaging issue in the Bragg peak. The feasibility of manufacturing this detector in smaller diameters will be the subject of further testing.

## References

[1] Bradley DA (2012). Review of doped silica glass optical fibre: Their TL properties and potential applications in radiation therapy dosimetry. Appl. Radiat. Isotop..

[2] Carrasco P (2015). Characterization of the Exradin W1 scintillator for use in radiotherapy. Medical Physics.

[3] Girard S (2017). Potential of Copper- and Cerium-doped Optical Fiber Materials for Proton Beam Monitoring. IEEE Trans. Nucl. Sci..

[4] Hoehr C (2018). Characterization of the Exradin W1 scintillator for 74 MeV proton therapy for ocular, applications. Physics in Medicine and Biology.

[5] OâĂŹKeeffe S (2015). A review of recent advances in optical fibre sensors for *in vivo* dosimetry during radiotherapy. Br J Radiol.

[6] Penner C (2017). Characterization of a Terbium Activated Gadolinium Oxysulfide Plastic Optical Fibre Sensor in Photons and Protons. IEEE Transactions on Optical Sensors.

[7] Veronese I (2010). Feasibility study for the use of cerium-doped silica fibres in proton therapy. Radiat Measurem.

[8] Veronese I (2017). Real-time dosimetry with yb-doped silica optical fibres. Physics in Medicine and Biology.

[9] Yanagida Y (2013). Study of rare-earth-doped scintillators. Opt. Mat..

[10] Torrisi L (2000). Plastic scintillator investigations for relative dosimetry in proton-therapy. Nucl. Instrum. Methods Phys. Res..

[11] Particle Therapy Co-Operative Group, http://www.ptcog.ch/ (accessed Dec. 19, 2018).

[12] Archambault L, Polf JC, Beaulieu L, Beddar S (2008). Characterizing the response of miniature scintillation detectors when irradiated with proton beams. Phys. Med. Biol..

[13] Girard S (2019). X-rays, γ-rays and Proton Beam Monitoring with Multimode Nitrogen-doped Optical Fiber. IEEE TNS.

[14] Hoehr, C. *et al*. Potential of Novel Optical Fibers for Proton Therapy Dosimetry. IEEE Nuclear Science Symposium and Medical Imaging Conference, (Atlanta, United States, Oct 2017).

[15] Savard N (2018). Characteristic of a Ce-Doped silica fiber irradiated by 74 MeV protons. Radiation Measurements.

[16] Wootton L, Holmes C, Sahoo N, Beddar S (2014). Passively scattered proton beam entrance dosimetry with a plastic scintillation detector. Physics in Medicine and Biology.

[17] Brage J, Andersen C, Christensen C, Christensen C (2018). Relating ionization quenching in organic plastic scintillators to basic material properties by modelling excitation density transport and amorphous track structure during proton irradiation. Physics in Medicine and Biology.

[18] El Hamzaoui H (2019). Gd^3+^ -doped sol-gel silica glass for remote ionizing radiation dosimetry. OSA Contrinuum.

[19] El Hamzaoui H (2010). From porous silica xerogels to bulk optical glasses: The control of densification. Mater. Chem. Phys..

[20] El Hamzaoui H (2012). Sol-gel derived ionic copper-doped microstructured optical fiber: a potential selective ultraviolet radiation dosimeter. Opt. Express.

[21] El Hamzaoui H (2014). Effects of densification atmosphere on optical properties of ionic copper-activated sol-gel silica glass: towards an efficient radiation dosimeter. Mater. Res. Express.

[22] El Hamzaoui H (2016). Cerium-activated sol-gel silica glasses for radiation dosimetry in harsh environment. Mater. Res. Express.

[23] Capoen B (2016). SolâĂŞgel derived copper-doped silica glass as a sensitive material for X-ray beam dosimetry. Opt. Mater..

[24] Blackmore, E., Evans, B.& Mouat, M.Operation of the TRIUMF Proton Therapy Facility. PAC’97 Conference Proceedings, 3831–3833 (1997).

[25] Tran E, Ma R, Paton K, Blackmore E, Pickles T (2012). Outcomes of Proton Therapy for peripapillary Choroidal Melanoma at the BC Cancer Agency. Int. J. Radiat. Oncol. BioI. Phys..

[26] Weber B, Paton K, Ma R, Pickles T (2015). 2015 Outcomes of Proton Beam Radiotherapy for Large Non-Peripapillary Choroidal and Ciliary Body Melanoma at TRIUMF and the BC Cancer Agency. Ocular Oncol. Pathol..

[27] Hrbacek J (2016). Practice Patterns Analysis of Ocular Proton Therapy Centers: The International OPTIC Survey. International Journal of Radiation Oncology Biology Physics.

[28] Bylinskii I, Craddock MK (2014). The TRIUMF 500 MeV cyclotron: the driver accelerator. Hyperfine Interactions.

[29] Absorbed Dose Determination in External Beam Radiotherapy: An International Code of Practice for Dosimetry Based on Standards of Absorbed Dose to Water, Technical Report Series no 398 (Vienna: IAEA) (IAEA, 2000).

[30] Birks, J. B.*The Theory and Practice of Scintillation Counting* (Oxford: Pergamon, 1964).

[31] Chou CN (1952). The nature of the saturation effect of fluorescent scintillators. Phys. Rev..

[32] Craun RL, Smith DL (1970). Analysis of response data for several organic scintillators. Nucl. Instrum. Methods.

[33] Ziegler J, Ziegler M, Biersack J (2010). SRIM - The stopping and range of ions in matter (2010). Nucl. Instrum. Methods B.

[34] Alonso PJ, Orera VM, Cases R, Alcala R, Rodriguez VD (1988). Optical properties of Gd^3+^ in fluorozirconate glasses. J. Lumin..

[35] Binnemans K, GÃűrller-Walrand C, Adam JL (1997). Spectroscopic properties of Gd^3+^-doped fluorozirconate glass. Chem. Phys. Lett..

[36] Mace H, Reisfeld R, David Avnir D (1983). Fluorescence of rare earth ions adsorbed on porous vycor glass. Chem. Phys. Lett..

[37] Caspers HH, Miller SA, Rast HE, Fry JL (1969). Electronic Spectrum and Energy Levels of Gd^3+^ in LaF3. Phys. Rev..

[38] Wybourne BG (1966). Energy Levels of Trivalent Gadolinium and Ionic Contributions to the Ground-State Splitting. Phys. Rev..

[39] Doran S (2015). Issues involved in the quantitative 3D imaging of proton doses using optical CT and chemical dosimeters. Phys. Med. Biol..

[40] Jiménez JA (2016). What is the origin of concentration quenching of Cu^+^ luminescence in glass?. Physica B: Condensed Matter..

[41] Sontakke AD, Ueda J, Tanabe S (2016). Effect of synthesis conditions on Ce^3+^ luminescence in borate glasses. J. Non-Cryst. Solids.

[42] Beckurts, K. H.& Wirtz, K. *Neutron Physics*, Germany, (Berlin:Springer-Verlag, 1964).

